# Supplementary material for: Altered microRNA Transcriptome in Cultured Human Liver Cells upon Infection with Ebola Virus
Source: Int J Mol Sci. 2021 Apr 6;22(7):3792. doi: 10.3390/ijms22073792 (PMC8038836; doi:10.3390/ijms22073792)
Supplement: Supplementary file 1 [file ijms-22-03792-s001.zip › Supplementary_File/C_ GO_Analysis_Results/16-30nt_go_Reston-24h-Huh7_vs_Control-24h-Huh7_down.mature_mirna_targets/CC_result(Human).html]

| GO.ID | Term | Ontology | Count | Pop.Hits | List.Total | Pop.Total | Fold.Enrichment | Pvalue | FDR | Enrichment.Score | Gene.Ratio | GENES |
| --- | --- | --- | --- | --- | --- | --- | --- | --- | --- | --- | --- | --- |
| GO:0012505 | endomembrane system | Cellular component | 62 | 4421 | 181 | 18698 | 1.44873100633466 | 0.000768875593507436 | 0.309684069369345 | 3.11414392472917 | 0.342541436464088 | CBX5//STMN2//STEAP2//RHOA//ANKRD50//CHP1//ZDHHC19//ZDHHC20//SLC37A4//AMFR//ASPH//UFM1//NDFIP2//STIM1//THY1//FA2H//TMX1//AOC3//ZW10//EI24//SCAMP2//CDIPT//BAIAP2//FGD1//CAMSAP2//TXNDC8//RAB2A//VIPAS39//LZTR1//KDELR1//VAMP2//NF2//ST8SIA2//MAP1LC3A//ARHGAP1//CDK16//DNAJC5//DOC2A//WDR3//TMEM201//NDUFB4//STARD3//CD300LG//HLA-DPA1//SIRPB1//CR1//RAB9B//SYT2//SLC17A5//XYLT2//MOSPD3//RNF26//MAPK8IP1//LRG1//CSF1//IGFBP7//ADAMTSL4//SMPD3//ARF3//HS3ST3B1//TBC1D9B//PITPNM2// |
| GO:0030133 | transport vesicle | Cellular component | 11 | 381 | 181 | 18698 | 2.98252635547628 | 0.00124902007821076 | 0.309684069369345 | 2.90343058021189 | 0.0607734806629834 | HLA-DPA1//STEAP2//DOC2A//CDK16//VAMP2//DNAJC5//SYT2//SLC17A5//SCAMP2//KDELR1//CHP1// |
| GO:0005622 | intracellular | Cellular component | 158 | 14673 | 181 | 18698 | 1.11238404209935 | 0.0015498937799043 | 0.309684069369345 | 2.8096980646569 | 0.87292817679558 | CBX5//HDAC11//TBL1Y//SMPD3//CDIPT//KDELR1//CHP1//HLA-DPA1//ARF3//NDFIP2//RAB2A//XYLT2//ST8SIA2//HS3ST3B1//KIF18B//GNA14//MAP1LC3A//PSMC6//ZW10//PRM1//GABPA//POU4F1//GATAD2B//ANP32E//TMEM201//AGO1//SAMD4B//GRPEL2//TXNDC8//TRIM29//SLC17A5//DOC2A//STARD3//CKMT2//CS//SLC9B1//THEM5//RHOA//NDUFB4//PCCB//PRODH//MAVS//NCOA4//DNAJC5//ARPC3//VAV3//BAIAP2//KLHL2//PIK3AP1//SRXN1//C8ORF37//ETF1//FGD1//PDZRN3//CAMSAP2//STEAP2//IARS//KRT13//ARHGAP1//MTAP//NF2//RAB9B//CDK16//PFKFB3//WWC3//PITPNM2//RANBP10//DDX55//ABCD3//LHPP//VAMP2//THY1//CDK2AP1//RGS5//PEA15//FBP2//MAPK8IP1//EEF1E1//EI24//WDR3//PPM1M//ZBTB46//PPP4R2//ZNF609//SPDEF//FAM32A//NR6A1//AMFR//PROX2//HLF//MYPOP//UFM1//ANKRD49//ZC4H2//ZNF490//RIT2//SNAPC1//TEAD3//VDR//ZNF24//IFRD2//ERI1//ZNF682//IFI44//PIM2//STMN2//PRDM4//MORN4//METTL21A//DNAH12//CTNNA3//MAP3K9//ATP1B2//MYO15A//SGTB//VIPAS39//DEPTOR//TNFAIP8L2//DPF1//AOC3//SNRNP27//POLI//ESRRG//MAFG//DDA1//MORC4//ZNF672//SLC25A45//ANKRD50//ZDHHC19//ZDHHC20//SLC37A4//ASPH//STIM1//FA2H//TMX1//CSF1//IGFBP7//ADAMTSL4//MOSPD3//RNF26//SCAMP2//LRG1//FXYD2//LZTR1//SIRPB1//CR1//TPPP3//LRP8//FAM206A//SYT2//GRIA3//CD300LG//TOM1L2//TBC1D9B//MAS1//TRIM44//SIRPG// |
| GO:0043227 | membrane-bounded organelle | Cellular component | 140 | 12616 | 181 | 18698 | 1.14636504727838 | 0.00221571002738551 | 0.309684069369345 | 2.65448707686173 | 0.773480662983425 | SCAMP2//LRG1//HLA-DPA1//RHOA//FXYD2//NDFIP2//ABCD3//VAMP2//FA2H//MAP1LC3A//STARD3//CKMT2//GRPEL2//CS//SLC9B1//THEM5//NDUFB4//PCCB//PRODH//MAVS//NCOA4//DNAJC5//WDR3//CHP1//PPM1M//ZBTB46//KIF18B//PPP4R2//PDZRN3//ZNF609//CBX5//GABPA//SPDEF//FAM32A//NR6A1//AGO1//AMFR//PROX2//HLF//MYPOP//KRT13//MTAP//NF2//CDK16//UFM1//POU4F1//ANKRD49//SAMD4B//ZC4H2//PSMC6//ZNF490//RANBP10//DDX55//RAB2A//RIT2//LHPP//SNAPC1//TEAD3//VDR//ZNF24//IFRD2//HDAC11//CDK2AP1//ANP32E//DOC2A//PEA15//ERI1//ZNF682//ZW10//MAPK8IP1//EEF1E1//ZDHHC19//ZDHHC20//SLC37A4//ASPH//STIM1//THY1//TMX1//AOC3//EI24//KDELR1//CDIPT//BAIAP2//STMN2//FGD1//CAMSAP2//TXNDC8//VIPAS39//LZTR1//TRIM29//SLC17A5//ARPC3//SIRPB1//VAV3//SLAMF6//CR1//CSF1//TOM1L2//CD300LG//BROX//EFNA1//GYPA//IARS//IGFBP7//ARF3//ARHGAP1//MYO15A//TPPP3//ABHD8//FBP2//GNA14//STEAP2//ANKRD50//RAB9B//ST8SIA2//GRIA3//SYT2//SMPD3//XYLT2//HS3ST3B1//SLC25A45//TMEM201//TBL1Y//GATAD2B//SNRNP27//POLI//METTL21A//ESRRG//MAFG//PFKFB3//PRM1//DDA1//MORC4//ZNF672//FAM206A//PRDM4//DPF1//ADAMTSL4//MOSPD3//RNF26// |
| GO:0030027 | lamellipodium | Cellular component | 7 | 187 | 181 | 18698 | 3.86698968889414 | 0.00233319067744021 | 0.309684069369345 | 2.63204976751734 | 0.0386740331491713 | ARPC3//STMN2//KLHL2//FGD1//CTNNA3//RHOA//NF2// |
| GO:1904949 | ATPase complex | Cellular component | 5 | 97 | 181 | 18698 | 5.32494161872757 | 0.00252752057000901 | 0.309684069369345 | 2.59730530125425 | 0.0276243093922652 | DPF1//ANP32E//GATAD2B//ATP1B2//FXYD2// |
| GO:0005794 | Golgi apparatus | Cellular component | 26 | 1501 | 181 | 18698 | 1.78940743003743 | 0.00267361227781358 | 0.309684069369345 | 2.57290157307066 | 0.143646408839779 | SMPD3//CDIPT//KDELR1//CHP1//HLA-DPA1//ARF3//NDFIP2//RAB2A//XYLT2//ST8SIA2//HS3ST3B1//VAMP2//STEAP2//SCAMP2//BAIAP2//STMN2//ZDHHC19//FGD1//CAMSAP2//ZDHHC20//TXNDC8//AMFR//VIPAS39//LZTR1//AOC3//EI24// |
| GO:0000118 | histone deacetylase complex | Cellular component | 4 | 61 | 181 | 18698 | 6.77402409202065 | 0.0028841356867925 | 0.309684069369345 | 2.53998431169218 | 0.0220994475138122 | GATAD2B//CBX5//HDAC11//TBL1Y// |
| GO:0031982 | vesicle | Cellular component | 58 | 4372 | 181 | 18698 | 1.37045386765605 | 0.00467380712833856 | 0.354062660050449 | 2.33032921338722 | 0.320441988950276 | RHOA//VAMP2//ANP32E//SCAMP2//ARPC3//SIRPB1//VAV3//BAIAP2//CHP1//SLAMF6//LRG1//CR1//CS//CSF1//TOM1L2//CD300LG//BROX//EFNA1//TXNDC8//GYPA//IARS//IGFBP7//ARF3//KRT13//ARHGAP1//MTAP//NDUFB4//FXYD2//MYO15A//UFM1//TPPP3//PSMC6//RAB2A//THY1//ABHD8//HDAC11//DNAJC5//FBP2//EEF1E1//GNA14//STMN2//STEAP2//ANKRD50//NF2//VIPAS39//ST8SIA2//AOC3//MAP1LC3A//KDELR1//RAB9B//DOC2A//CDK16//STARD3//NDFIP2//HLA-DPA1//GRIA3//SYT2//SLC17A5// |
| GO:0005890 | sodium:potassium-exchanging ATPase complex | Cellular component | 2 | 11 | 181 | 18698 | 18.7825213460573 | 0.00483944312404562 | 0.354062660050449 | 2.31520460985731 | 0.0110497237569061 | ATP1B2//FXYD2// |
| GO:0032585 | multivesicular body membrane | Cellular component | 2 | 11 | 181 | 18698 | 18.7825213460573 | 0.00483944312404562 | 0.354062660050449 | 2.31520460985731 | 0.0110497237569061 | CD300LG//NDFIP2// |
| GO:0031984 | organelle subcompartment | Cellular component | 27 | 1655 | 181 | 18698 | 1.68531989117191 | 0.00501281230677175 | 0.354062660050449 | 2.2999185562406 | 0.149171270718232 | VAMP2//SMPD3//ASPH//STIM1//SCAMP2//HLA-DPA1//CDIPT//KDELR1//CHP1//ARF3//NDFIP2//RAB2A//XYLT2//ST8SIA2//HS3ST3B1//STARD3//SLC37A4//AMFR//RHOA//MOSPD3//RNF26//FA2H//TMX1//ZW10//MAPK8IP1//EI24//STEAP2// |
| GO:0017101 | aminoacyl-tRNA synthetase multienzyme complex | Cellular component | 2 | 12 | 181 | 18698 | 17.2173112338858 | 0.00577052065274305 | 0.354062660050449 | 2.23878500029104 | 0.0110497237569061 | IARS//EEF1E1// |
| GO:0060198 | clathrin-sculpted vesicle | Cellular component | 2 | 12 | 181 | 18698 | 17.2173112338858 | 0.00577052065274305 | 0.354062660050449 | 2.23878500029104 | 0.0110497237569061 | VAMP2//DNAJC5// |
| GO:0005783 | endoplasmic reticulum | Cellular component | 29 | 1859 | 181 | 18698 | 1.6115181036558 | 0.00675110535140695 | 0.355169139399321 | 2.17062511476667 | 0.160220994475138 | BAIAP2//ASPH//STIM1//CSF1//IGFBP7//ADAMTSL4//CDIPT//KDELR1//STARD3//SLC37A4//AMFR//RHOA//RAB2A//XYLT2//MOSPD3//RNF26//FA2H//TMX1//ZW10//MAPK8IP1//EI24//HLA-DPA1//CHP1//ZDHHC19//ZDHHC20//UFM1//NDFIP2//THY1//AOC3// |
| GO:0098588 | bounding membrane of organelle | Cellular component | 31 | 2040 | 181 | 18698 | 1.56981367132488 | 0.00738489957529858 | 0.355169139399321 | 2.1316554061327 | 0.171270718232044 | CDIPT//KDELR1//CHP1//HLA-DPA1//ARF3//NDFIP2//SMPD3//RAB2A//XYLT2//ST8SIA2//HS3ST3B1//SIRPB1//CR1//RHOA//RAB9B//VAMP2//STEAP2//ARHGAP1//GRIA3//ASPH//STIM1//SCAMP2//MAP1LC3A//DNAJC5//SYT2//STARD3//MAVS//ABCD3//SLC17A5//DOC2A//CD300LG// |
| GO:0008021 | synaptic vesicle | Cellular component | 6 | 176 | 181 | 18698 | 3.52172275238574 | 0.00740946311356997 | 0.355169139399321 | 2.13021325966728 | 0.0331491712707182 | SYT2//SLC17A5//VAMP2//DOC2A//CDK16//DNAJC5// |
| GO:0044424 | intracellular part | Cellular component | 153 | 14395 | 181 | 18698 | 1.09798483589491 | 0.00779615317550059 | 0.355169139399321 | 2.10811963663739 | 0.845303867403315 | GNA14//PSMC6//BAIAP2//IFI44//STARD3//PIM2//STMN2//PRDM4//CHP1//KLHL2//MORN4//SRXN1//KIF18B//METTL21A//PPP4R2//C8ORF37//TMEM201//DNAH12//ETF1//FGD1//TXNDC8//AGO1//CTNNA3//IARS//RHOA//ARHGAP1//MAP3K9//MTAP//NF2//ATP1B2//MYO15A//CDK16//UFM1//SGTB//POU4F1//NDFIP2//ZC4H2//DDX55//RIT2//VIPAS39//DEPTOR//VDR//TNFAIP8L2//DPF1//RGS5//AOC3//FBP2//ERI1//MAPK8IP1//EEF1E1//NR6A1//TEAD3//CKMT2//SLC25A45//NDUFB4//PRODH//ABCD3//MAVS//CBX5//SCAMP2//LRG1//HLA-DPA1//FXYD2//VAMP2//GRPEL2//CS//SLC9B1//THEM5//PCCB//NCOA4//DNAJC5//ARPC3//VAV3//PIK3AP1//PDZRN3//CAMSAP2//STEAP2//SLC17A5//KRT13//RAB9B//PFKFB3//SAMD4B//WWC3//PITPNM2//RANBP10//RAB2A//LHPP//THY1//CDK2AP1//MAP1LC3A//PEA15//ZW10//EI24//ZDHHC19//ZDHHC20//SLC37A4//AMFR//ASPH//STIM1//FA2H//TMX1//KDELR1//CDIPT//LZTR1//ANP32E//POLI//CSF1//ARF3//TPPP3//IGFBP7//ADAMTSL4//DDA1//TRIM29//DOC2A//SNAPC1//WDR3//PPM1M//ZBTB46//ZNF609//GABPA//SPDEF//FAM32A//PROX2//HLF//MYPOP//ANKRD49//ZNF490//ZNF24//IFRD2//HDAC11//ZNF682//ANKRD50//ST8SIA2//PRM1//GATAD2B//TBL1Y//SNRNP27//ESRRG//MAFG//MORC4//ZNF672//LRP8//SMPD3//XYLT2//HS3ST3B1//MOSPD3//RNF26//SIRPB1//CR1//GRIA3//SYT2//CD300LG//FAM206A// |
| GO:0005767 | secondary lysosome | Cellular component | 2 | 14 | 181 | 18698 | 14.7576953433307 | 0.00785589481791281 | 0.355169139399321 | 2.10480433992399 | 0.0110497237569061 | NCOA4//MAP1LC3A// |
| GO:0043226 | organelle | Cellular component | 146 | 13597 | 181 | 18698 | 1.10924208581922 | 0.00835041750823485 | 0.358650431978687 | 2.07829180990687 | 0.806629834254144 | DNAH12//SCAMP2//LRG1//HLA-DPA1//RHOA//FXYD2//NDFIP2//ABCD3//VAMP2//BAIAP2//LRP8//STARD3//CKMT2//GRPEL2//CS//SLC9B1//THEM5//NDUFB4//PCCB//PRODH//MAVS//NCOA4//DNAJC5//WDR3//CHP1//PPM1M//ZBTB46//KIF18B//PPP4R2//PDZRN3//ZNF609//CBX5//GABPA//SPDEF//FAM32A//NR6A1//AGO1//AMFR//PROX2//HLF//MYPOP//KRT13//MTAP//NF2//CDK16//UFM1//POU4F1//ANKRD49//SAMD4B//ZC4H2//PSMC6//ZNF490//RANBP10//DDX55//RAB2A//RIT2//LHPP//SNAPC1//TEAD3//VDR//ZNF24//IFRD2//HDAC11//CDK2AP1//ANP32E//DOC2A//PEA15//ERI1//ZNF682//ZW10//MAPK8IP1//EEF1E1//MAP1LC3A//ZDHHC19//ZDHHC20//SLC37A4//ASPH//STIM1//THY1//FA2H//TMX1//AOC3//EI24//KDELR1//CDIPT//STMN2//FGD1//CAMSAP2//TXNDC8//VIPAS39//LZTR1//ARPC3//SIRPB1//VAV3//SLAMF6//CR1//CSF1//TOM1L2//CD300LG//BROX//EFNA1//GYPA//IARS//IGFBP7//ARF3//ARHGAP1//MYO15A//TPPP3//ABHD8//FBP2//GNA14//CTNNA3//KLHL2//POLI//STEAP2//ANKRD50//RAB9B//TRIM29//SLC17A5//ST8SIA2//GRIA3//TMEM201//SYT2//TBL1Y//SMPD3//XYLT2//HS3ST3B1//PRM1//GATAD2B//SNRNP27//METTL21A//ESRRG//MAFG//PFKFB3//DDA1//MORC4//ZNF672//SLC25A45//ADAMTSL4//MOSPD3//RNF26//FAM206A//MORN4//PRDM4//DPF1//C8ORF37// |
| GO:0090533 | cation-transporting ATPase complex | Cellular component | 2 | 15 | 181 | 18698 | 13.7738489871087 | 0.00900717111312914 | 0.364358403156067 | 2.04541158669758 | 0.0110497237569061 | ATP1B2//FXYD2// |
| GO:0031090 | organelle membrane | Cellular component | 41 | 2950 | 181 | 18698 | 1.43574866560539 | 0.00933164711226248 | 0.364358403156067 | 2.03004169294889 | 0.226519337016575 | WDR3//TMEM201//NDUFB4//EI24//MAVS//MAPK8IP1//CKMT2//SLC25A45//PRODH//ABCD3//CDIPT//KDELR1//CHP1//HLA-DPA1//ARF3//NDFIP2//SMPD3//RAB2A//XYLT2//ST8SIA2//HS3ST3B1//SIRPB1//CR1//RHOA//RAB9B//VAMP2//STEAP2//ARHGAP1//GRIA3//ASPH//STIM1//SCAMP2//MAP1LC3A//STARD3//SLC17A5//DNAJC5//SYT2//DOC2A//CD300LG//GRPEL2//FA2H// |
| GO:0070382 | exocytic vesicle | Cellular component | 6 | 190 | 181 | 18698 | 3.26222739168363 | 0.0105761430997508 | 0.372151034943672 | 1.97567268162813 | 0.0331491712707182 | CDK16//VAMP2//DNAJC5//SYT2//SLC17A5//DOC2A// |
| GO:0070062 | extracellular exosome | Cellular component | 39 | 2817 | 181 | 18698 | 1.43019198747933 | 0.0120441861589125 | 0.372151034943672 | 1.91922254050869 | 0.215469613259669 | SCAMP2//ARPC3//SIRPB1//VAV3//BAIAP2//CHP1//SLAMF6//LRG1//CR1//CS//CSF1//TOM1L2//CD300LG//BROX//EFNA1//TXNDC8//GYPA//IARS//IGFBP7//ARF3//KRT13//RHOA//ARHGAP1//MTAP//NDUFB4//FXYD2//MYO15A//UFM1//TPPP3//PSMC6//RAB2A//VAMP2//THY1//ABHD8//HDAC11//DNAJC5//FBP2//EEF1E1//GNA14// |
| GO:0031252 | cell leading edge | Cellular component | 9 | 385 | 181 | 18698 | 2.41489560163593 | 0.0128834251432918 | 0.372151034943672 | 1.88996866158178 | 0.0497237569060773 | BAIAP2//KLHL2//FGD1//ARPC3//STMN2//CTNNA3//RHOA//NF2//THY1// |
| GO:0097458 | neuron part | Cellular component | 24 | 1533 | 181 | 18698 | 1.6172816814609 | 0.0130648062758723 | 0.372151034943672 | 1.88389702558024 | 0.132596685082873 | CDK16//VAMP2//DNAJC5//BAIAP2//LRP8//STMN2//RHOA//NF2//POU4F1//RIT2//DOC2A//SYT2//SLC17A5//MORN4//THY1//AMFR//DPF1//GRIA3//CADM2//FAM206A//MYO15A//CNTNAP1//ARPC3//MAPK8IP1// |
| GO:0005789 | endoplasmic reticulum membrane | Cellular component | 18 | 1050 | 181 | 18698 | 1.77092344119968 | 0.013389785082318 | 0.372151034943672 | 1.87322639373575 | 0.0994475138121547 | ASPH//STIM1//AMFR//SLC37A4//HLA-DPA1//CDIPT//KDELR1//STARD3//RHOA//RAB2A//XYLT2//MOSPD3//RNF26//FA2H//TMX1//ZW10//MAPK8IP1//EI24// |
| GO:1903561 | extracellular vesicle | Cellular component | 39 | 2837 | 181 | 18698 | 1.42010956247067 | 0.0134417765028937 | 0.372151034943672 | 1.8715433299118 | 0.215469613259669 | SCAMP2//ARPC3//SIRPB1//VAV3//BAIAP2//CHP1//SLAMF6//LRG1//CR1//CS//CSF1//TOM1L2//CD300LG//BROX//EFNA1//TXNDC8//GYPA//IARS//IGFBP7//ARF3//KRT13//RHOA//ARHGAP1//MTAP//NDUFB4//FXYD2//MYO15A//UFM1//TPPP3//PSMC6//RAB2A//VAMP2//THY1//ABHD8//HDAC11//DNAJC5//FBP2//EEF1E1//GNA14// |
| GO:0043230 | extracellular organelle | Cellular component | 39 | 2839 | 181 | 18698 | 1.41910913305012 | 0.0135886165245821 | 0.372151034943672 | 1.8668247571251 | 0.215469613259669 | SCAMP2//ARPC3//SIRPB1//VAV3//BAIAP2//CHP1//SLAMF6//LRG1//CR1//CS//CSF1//TOM1L2//CD300LG//BROX//EFNA1//TXNDC8//GYPA//IARS//IGFBP7//ARF3//KRT13//RHOA//ARHGAP1//MTAP//NDUFB4//FXYD2//MYO15A//UFM1//TPPP3//PSMC6//RAB2A//VAMP2//THY1//ABHD8//HDAC11//DNAJC5//FBP2//EEF1E1//GNA14// |
| GO:0098827 | endoplasmic reticulum subcompartment | Cellular component | 18 | 1054 | 181 | 18698 | 1.76420266912682 | 0.0138728446634128 | 0.372151034943672 | 1.85783447656542 | 0.0994475138121547 | CDIPT//KDELR1//STARD3//SLC37A4//AMFR//RHOA//ASPH//RAB2A//XYLT2//MOSPD3//STIM1//RNF26//FA2H//TMX1//ZW10//MAPK8IP1//EI24//HLA-DPA1// |
| GO:0043231 | intracellular membrane-bounded organelle | Cellular component | 120 | 10864 | 181 | 18698 | 1.14105891829877 | 0.0142918993046559 | 0.372151034943672 | 1.8449100523343 | 0.662983425414365 | STARD3//CKMT2//GRPEL2//CS//SLC9B1//THEM5//RHOA//NDUFB4//PCCB//NDFIP2//PRODH//MAVS//NCOA4//DNAJC5//WDR3//CHP1//PPM1M//ZBTB46//KIF18B//PPP4R2//PDZRN3//ZNF609//CBX5//GABPA//SPDEF//FAM32A//NR6A1//AGO1//AMFR//PROX2//HLF//MYPOP//KRT13//MTAP//NF2//CDK16//UFM1//POU4F1//ANKRD49//SAMD4B//ZC4H2//PSMC6//ZNF490//RANBP10//DDX55//RAB2A//RIT2//LHPP//SNAPC1//TEAD3//VDR//ZNF24//IFRD2//HDAC11//CDK2AP1//ANP32E//DOC2A//PEA15//ERI1//ZNF682//ZW10//MAPK8IP1//EEF1E1//ZDHHC19//ZDHHC20//SLC37A4//ASPH//STIM1//THY1//FA2H//TMX1//AOC3//EI24//KDELR1//SCAMP2//CDIPT//BAIAP2//STMN2//FGD1//CAMSAP2//TXNDC8//VIPAS39//LZTR1//MAP1LC3A//ABCD3//TRIM29//SLC17A5//HLA-DPA1//SLC25A45//TBL1Y//GATAD2B//TMEM201//SNRNP27//POLI//METTL21A//ESRRG//MAFG//PFKFB3//PRM1//DDA1//MORC4//ZNF672//FBP2//FAM206A//PRDM4//DPF1//CSF1//IGFBP7//ADAMTSL4//XYLT2//MOSPD3//RNF26//SMPD3//ARF3//ST8SIA2//HS3ST3B1//VAMP2//STEAP2//LRG1//FXYD2// |
| GO:0098805 | whole membrane | Cellular component | 25 | 1630 | 181 | 18698 | 1.58441514422262 | 0.0143996165583113 | 0.372151034943672 | 1.8416490724066 | 0.138121546961326 | HLA-DPA1//SIRPB1//CR1//RHOA//RAB9B//VAMP2//STEAP2//ARHGAP1//GRIA3//MAP1LC3A//DNAJC5//SYT2//STARD3//SCAMP2//KDELR1//MAVS//ABCD3//SLC17A5//RAB2A//DOC2A//CD300LG//NDFIP2//LRP8//RIT2//THY1// |
| GO:0043005 | neuron projection | Cellular component | 20 | 1224 | 181 | 18698 | 1.68797168959665 | 0.0151062089243085 | 0.372151034943672 | 1.82084451314826 | 0.110497237569061 | CADM2//RHOA//LRP8//AMFR//FAM206A//THY1//MYO15A//BAIAP2//VAMP2//RIT2//MAPK8IP1//MORN4//STMN2//CNTNAP1//DNAJC5//ARPC3//NF2//CDK16//POU4F1//DOC2A// |
| GO:0030672 | synaptic vesicle membrane | Cellular component | 4 | 98 | 181 | 18698 | 4.21648438380877 | 0.0151633134144686 | 0.372151034943672 | 1.81920588838819 | 0.0220994475138122 | SYT2//SLC17A5//VAMP2//DOC2A// |
| GO:0099501 | exocytic vesicle membrane | Cellular component | 4 | 98 | 181 | 18698 | 4.21648438380877 | 0.0151633134144686 | 0.372151034943672 | 1.81920588838819 | 0.0220994475138122 | SYT2//SLC17A5//VAMP2//DOC2A// |
| GO:0071782 | endoplasmic reticulum tubular network | Cellular component | 2 | 20 | 181 | 18698 | 10.3303867403315 | 0.0157909959423286 | 0.373352000577 | 1.801590478059 | 0.0110497237569061 | ASPH//STIM1// |
| GO:0042175 | nuclear outer membrane-endoplasmic reticulum membrane network | Cellular component | 18 | 1071 | 181 | 18698 | 1.73619945215655 | 0.0160815180690908 | 0.373352000577 | 1.7936729569613 | 0.0994475138121547 | CDIPT//KDELR1//STARD3//SLC37A4//AMFR//RHOA//ASPH//RAB2A//XYLT2//MOSPD3//STIM1//RNF26//FA2H//TMX1//ZW10//MAPK8IP1//EI24//HLA-DPA1// |
| GO:0044432 | endoplasmic reticulum part | Cellular component | 21 | 1319 | 181 | 18698 | 1.64471661521578 | 0.0167941811116997 | 0.376898181654847 | 1.77484116760919 | 0.116022099447514 | CSF1//IGFBP7//ADAMTSL4//CDIPT//KDELR1//STARD3//SLC37A4//AMFR//RHOA//ASPH//RAB2A//XYLT2//MOSPD3//STIM1//RNF26//FA2H//TMX1//ZW10//MAPK8IP1//EI24//HLA-DPA1// |
| GO:0030426 | growth cone | Cellular component | 5 | 154 | 181 | 18698 | 3.3540216689388 | 0.0171117917165763 | 0.376898181654847 | 1.76670451460367 | 0.0276243093922652 | MAPK8IP1//ARPC3//STMN2//AMFR//THY1// |
| GO:0030054 | cell junction | Cellular component | 20 | 1247 | 181 | 18698 | 1.65683829034988 | 0.0181189161685868 | 0.38183089611488 | 1.74186778436781 | 0.110497237569061 | BAIAP2//TRIM29//CADM2//CNTNAP1//RHOA//NF2//CTNNA3//ARPC3//CHP1//THY1//SYT2//C8ORF37//PDZRN3//SLC17A5//GRIA3//CDK16//ZC4H2//VAMP2//DOC2A//FBP2// |
| GO:0030665 | clathrin-coated vesicle membrane | Cellular component | 4 | 104 | 181 | 18698 | 3.97322566935827 | 0.0184655178388194 | 0.38183089611488 | 1.73363850867976 | 0.0220994475138122 | HLA-DPA1//VAMP2//DNAJC5//SYT2// |
| GO:0031410 | cytoplasmic vesicle | Cellular component | 32 | 2279 | 181 | 18698 | 1.45051503155159 | 0.0192272712880383 | 0.38183089611488 | 1.71608234595576 | 0.176795580110497 | STMN2//STEAP2//RHOA//ANKRD50//SCAMP2//KDELR1//CHP1//RAB9B//NF2//VIPAS39//ST8SIA2//AOC3//MAP1LC3A//ARHGAP1//DOC2A//VAMP2//RAB2A//DNAJC5//BAIAP2//CDK16//TXNDC8//STARD3//CD300LG//NDFIP2//HLA-DPA1//SIRPB1//CR1//GRIA3//SYT2//SLC17A5//LRG1//ANP32E// |
| GO:0030427 | site of polarized growth | Cellular component | 5 | 159 | 181 | 18698 | 3.24854928941242 | 0.0193655251126681 | 0.38183089611488 | 1.71297072225434 | 0.0276243093922652 | STMN2//AMFR//THY1//MAPK8IP1//ARPC3// |
| GO:0097708 | intracellular vesicle | Cellular component | 32 | 2282 | 181 | 18698 | 1.44860813186068 | 0.0195582764016935 | 0.38183089611488 | 1.70866942062285 | 0.176795580110497 | RHOA//VAMP2//ANP32E//STMN2//STEAP2//ANKRD50//SCAMP2//KDELR1//CHP1//RAB9B//NF2//VIPAS39//ST8SIA2//AOC3//MAP1LC3A//ARHGAP1//CDK16//DNAJC5//DOC2A//BAIAP2//RAB2A//TXNDC8//STARD3//CD300LG//NDFIP2//HLA-DPA1//SIRPB1//CR1//GRIA3//SYT2//SLC17A5//LRG1// |
| GO:0098533 | ATPase dependent transmembrane transport complex | Cellular component | 2 | 23 | 181 | 18698 | 8.9829449915926 | 0.0206327302250678 | 0.393855894740739 | 1.68544330022716 | 0.0110497237569061 | ATP1B2//FXYD2// |
| GO:0043229 | intracellular organelle | Cellular component | 135 | 12604 | 181 | 18698 | 1.10647588856296 | 0.0215900366892333 | 0.396968366985856 | 1.6657466196427 | 0.74585635359116 | SCAMP2//LRG1//HLA-DPA1//RHOA//FXYD2//NDFIP2//ABCD3//VAMP2//STARD3//CKMT2//GRPEL2//CS//SLC9B1//THEM5//NDUFB4//PCCB//PRODH//MAVS//NCOA4//DNAJC5//WDR3//CHP1//PPM1M//ZBTB46//KIF18B//PPP4R2//PDZRN3//ZNF609//CBX5//GABPA//SPDEF//FAM32A//NR6A1//AGO1//AMFR//PROX2//HLF//MYPOP//KRT13//MTAP//NF2//CDK16//UFM1//POU4F1//ANKRD49//SAMD4B//ZC4H2//PSMC6//ZNF490//RANBP10//DDX55//RAB2A//RIT2//LHPP//SNAPC1//TEAD3//VDR//ZNF24//IFRD2//HDAC11//CDK2AP1//ANP32E//DOC2A//PEA15//ERI1//ZNF682//ZW10//MAPK8IP1//EEF1E1//ZDHHC19//ZDHHC20//SLC37A4//ASPH//STIM1//THY1//FA2H//TMX1//AOC3//EI24//KDELR1//CDIPT//BAIAP2//STMN2//FGD1//CAMSAP2//TXNDC8//VIPAS39//LZTR1//CTNNA3//TRIM29//SLC17A5//MAP1LC3A//ARPC3//KLHL2//STEAP2//ANKRD50//ST8SIA2//RAB9B//ARHGAP1//POLI//CD300LG//SIRPB1//CR1//GRIA3//TMEM201//SYT2//MYO15A//DNAH12//TPPP3//LRP8//TBL1Y//SMPD3//ARF3//XYLT2//HS3ST3B1//PRM1//GATAD2B//SNRNP27//METTL21A//ESRRG//MAFG//PFKFB3//DDA1//MORC4//ZNF672//FBP2//SLC25A45//CSF1//IGFBP7//ADAMTSL4//MOSPD3//RNF26//FAM206A//PRDM4//DPF1// |
| GO:0001726 | ruffle | Cellular component | 5 | 164 | 181 | 18698 | 3.14950815254009 | 0.0218062230000098 | 0.396968366985856 | 1.66141955094145 | 0.0276243093922652 | RHOA//NF2//BAIAP2//KLHL2//FGD1// |
| GO:0005913 | cell-cell adherens junction | Cellular component | 4 | 110 | 181 | 18698 | 3.75650426921145 | 0.0221821671889652 | 0.396968366985856 | 1.65399602571481 | 0.0220994475138122 | CTNNA3//BAIAP2//TRIM29//CADM2// |
| GO:0044422 | organelle part | Cellular component | 104 | 9321 | 181 | 18698 | 1.15262334620156 | 0.0235773853233347 | 0.407598040135402 | 1.62750435880877 | 0.574585635359116 | FA2H//MAP1LC3A//MYO15A//MORN4//STARD3//WDR3//TMEM201//NDUFB4//EI24//MAVS//MAPK8IP1//SLC9B1//C8ORF37//FBP2//GRIA3//HLA-DPA1//SIRPB1//CR1//RHOA//RAB9B//VAMP2//CKMT2//SLC25A45//PRODH//ABCD3//CDIPT//KDELR1//CHP1//ARF3//NDFIP2//SMPD3//RAB2A//XYLT2//ST8SIA2//HS3ST3B1//STEAP2//ARHGAP1//ASPH//STIM1//CBX5//GRPEL2//CS//THEM5//PCCB//CSF1//IGFBP7//ADAMTSL4//LRG1//ZW10//PRM1//GABPA//POU4F1//GATAD2B//HDAC11//TBL1Y//BAIAP2//SNRNP27//POLI//METTL21A//PPP4R2//ESRRG//PDZRN3//ZNF609//NR6A1//AGO1//MAFG//PFKFB3//PSMC6//SNAPC1//TEAD3//VDR//DDA1//MORC4//ZNF672//CDK2AP1//ERI1//EEF1E1//FAM32A//NF2//DDX55//DOC2A//KIF18B//PRDM4//CAMSAP2//DNAH12//TPPP3//LRP8//PEA15//ARPC3//SCAMP2//SLC37A4//AMFR//MOSPD3//RNF26//TMX1//SYT2//DNAJC5//KRT13//SLC17A5//CD300LG//FAM206A//LHPP//ANP32E//DPF1// |
| GO:0005768 | endosome | Cellular component | 15 | 878 | 181 | 18698 | 1.76487244994274 | 0.0237251478542143 | 0.407598040135402 | 1.62479107238563 | 0.0828729281767956 | STEAP2//NF2//VIPAS39//ST8SIA2//AOC3//MAP1LC3A//ARHGAP1//STARD3//CD300LG//NDFIP2//SCAMP2//HLA-DPA1//STMN2//RHOA//ANKRD50// |
| GO:0000139 | Golgi membrane | Cellular component | 13 | 729 | 181 | 18698 | 1.84218144889313 | 0.0252676273540101 | 0.413731830984646 | 1.59743553668864 | 0.0718232044198895 | SCAMP2//HLA-DPA1//STEAP2//CDIPT//KDELR1//CHP1//ARF3//NDFIP2//SMPD3//RAB2A//XYLT2//ST8SIA2//HS3ST3B1// |
| GO:0030662 | coated vesicle membrane | Cellular component | 5 | 171 | 181 | 18698 | 3.02058091822558 | 0.0255469246773006 | 0.413731830984646 | 1.59266137247895 | 0.0276243093922652 | HLA-DPA1//KDELR1//SYT2//VAMP2//DNAJC5// |
| GO:0120025 | plasma membrane bounded cell projection | Cellular component | 28 | 1980 | 181 | 18698 | 1.46086277136001 | 0.0259853956551484 | 0.413731830984646 | 1.58527066619769 | 0.154696132596685 | BAIAP2//KLHL2//FGD1//DNAH12//STMN2//RHOA//NF2//CDK16//POU4F1//RIT2//VAMP2//DOC2A//ARPC3//CTNNA3//CADM2//LRP8//AMFR//FAM206A//THY1//MYO15A//AOC3//MAPK8IP1//TXNDC8//MORN4//SLC9B1//CNTNAP1//DNAJC5//C8ORF37// |
| GO:1990752 | microtubule end | Cellular component | 2 | 26 | 181 | 18698 | 7.94645133871653 | 0.0260087530537496 | 0.413731830984646 | 1.58488046882901 | 0.0110497237569061 | KIF18B//CAMSAP2// |
| GO:0098791 | Golgi subcompartment | Cellular component | 14 | 819 | 181 | 18698 | 1.76587807527034 | 0.0282954751211867 | 0.435495994422367 | 1.54828300924669 | 0.0773480662983425 | CDIPT//KDELR1//CHP1//HLA-DPA1//ARF3//NDFIP2//SMPD3//RAB2A//XYLT2//ST8SIA2//HS3ST3B1//VAMP2//SCAMP2//STEAP2// |
| GO:0030667 | secretory granule membrane | Cellular component | 7 | 303 | 181 | 18698 | 2.38655799281586 | 0.0283908913709576 | 0.435495994422367 | 1.54682097198943 | 0.0386740331491713 | DNAJC5//SYT2//VAMP2//CR1//RHOA//SIRPB1//RAB9B// |
| GO:0044446 | intracellular organelle part | Cellular component | 101 | 9080 | 181 | 18698 | 1.14908486869326 | 0.0297837897316199 | 0.44830545165192 | 1.52602004282242 | 0.558011049723757 | CBX5//ZW10//PRM1//BAIAP2//WDR3//SNRNP27//POLI//METTL21A//PPP4R2//ESRRG//PDZRN3//ZNF609//GABPA//NR6A1//AGO1//MAFG//PFKFB3//POU4F1//PSMC6//GATAD2B//SNAPC1//TEAD3//VDR//DDA1//MORC4//ZNF672//CDK2AP1//FBP2//ERI1//EEF1E1//FAM32A//NF2//DDX55//DOC2A//TMEM201//NDUFB4//EI24//GRPEL2//CS//THEM5//PCCB//PRODH//MAVS//MAPK8IP1//LRP8//PEA15//ARPC3//MYO15A//TPPP3//KIF18B//CAMSAP2//CDIPT//KDELR1//CHP1//HLA-DPA1//ARF3//NDFIP2//SMPD3//RAB2A//XYLT2//ST8SIA2//HS3ST3B1//CSF1//IGFBP7//ADAMTSL4//STARD3//SLC37A4//AMFR//RHOA//ASPH//MOSPD3//STIM1//RNF26//FA2H//TMX1//LRG1//SCAMP2//MAP1LC3A//SLC17A5//DNAJC5//ABCD3//DNAH12//SIRPB1//CR1//RAB9B//VAMP2//SYT2//GRIA3//STEAP2//CKMT2//SLC25A45//HDAC11//TBL1Y//PRDM4//ARHGAP1//FAM206A//LHPP//DPF1//KRT13//CD300LG//ANP32E// |
| GO:0030136 | clathrin-coated vesicle | Cellular component | 5 | 179 | 181 | 18698 | 2.88558288836075 | 0.030298621596316 | 0.44830545165192 | 1.51857712881554 | 0.0276243093922652 | STEAP2//SYT2//VAMP2//HLA-DPA1//DNAJC5// |
| GO:0044464 | cell part | Cellular component | 171 | 16886 | 181 | 18698 | 1.04613060085081 | 0.0307916433614241 | 0.44830545165192 | 1.51156713202072 | 0.94475138121547 | SIRPB1//CDIPT//VAV3//BAIAP2//CHP1//SLAMF6//PIK3AP1//SLC43A2//SYT2//CR1//CSF1//CD300LG//SLC9B1//C8ORF37//EFNA1//CADM2//ZDHHC20//STEAP2//SLC17A5//FFAR3//GRIA3//GYPA//HLA-DPA1//RHOA//MAS1//ASPH//NF2//ATP1B2//FXYD2//P2RY2//RAB9B//CDK16//SIRPG//SMPD3//SLC24A3//RIT2//SCN4A//STIM1//VAMP2//TECTB//THY1//LRP8//HDAC11//DNAJC5//DOC2A//RGS5//AOC3//FBP2//MAPK8IP1//GNA14//POLI//TOM1L2//TBC1D9B//TRIM44//DEPTOR//CLEC9A//PITPNM2//RAB2A//CNTNAP1//PPP4R2//STMN2//AMFR//ARPC3//PSMC6//IFI44//STARD3//PIM2//PRDM4//KLHL2//MORN4//SRXN1//KIF18B//METTL21A//TMEM201//DNAH12//ETF1//FGD1//TXNDC8//AGO1//CTNNA3//IARS//ARHGAP1//MAP3K9//MTAP//MYO15A//UFM1//SGTB//POU4F1//NDFIP2//ZC4H2//DDX55//VIPAS39//VDR//TNFAIP8L2//DPF1//ERI1//EEF1E1//NR6A1//TEAD3//VANGL1//NDUFB4//DDA1//SNAPC1//FAM206A//CKMT2//SLC25A45//PRODH//ABCD3//HS3ST3B1//CBX5//MAVS//ZW10//GRPEL2//CS//THEM5//PCCB//NCOA4//WDR3//PPM1M//ZBTB46//PDZRN3//ZNF609//GABPA//SPDEF//FAM32A//PROX2//HLF//MYPOP//KRT13//ANKRD49//SAMD4B//ZNF490//RANBP10//LHPP//ZNF24//IFRD2//CDK2AP1//ANP32E//PEA15//ZNF682//ZDHHC19//SLC37A4//FA2H//TMX1//EI24//SCAMP2//LRG1//KDELR1//CAMSAP2//LZTR1//PFKFB3//WWC3//MAP1LC3A//IGFBP7//ADAMTSL4//ARF3//TPPP3//XYLT2//ST8SIA2//ANKRD50//MOSPD3//RNF26//PRM1//SNRNP27//ESRRG//MAFG//GATAD2B//MORC4//ZNF672//TRIM29//TBL1Y// |
| GO:0070603 | SWI/SNF superfamily-type complex | Cellular component | 3 | 73 | 181 | 18698 | 4.24536441383486 | 0.0338708228596532 | 0.484917280607368 | 1.47017425245292 | 0.0165745856353591 | DPF1//GATAD2B//ANP32E// |
| GO:0005623 | cell | Cellular component | 171 | 16915 | 181 | 18698 | 1.04433705740271 | 0.0357161001410855 | 0.502952951167089 | 1.44713596802735 | 0.94475138121547 | SIRPB1//CDIPT//VAV3//BAIAP2//CHP1//SLAMF6//PIK3AP1//SLC43A2//SYT2//CR1//CSF1//CD300LG//SLC9B1//C8ORF37//EFNA1//CADM2//ZDHHC20//STEAP2//SLC17A5//FFAR3//GRIA3//GYPA//HLA-DPA1//RHOA//MAS1//ASPH//NF2//ATP1B2//FXYD2//P2RY2//RAB9B//CDK16//SIRPG//SMPD3//SLC24A3//RIT2//SCN4A//STIM1//VAMP2//TECTB//THY1//LRP8//HDAC11//DNAJC5//DOC2A//RGS5//AOC3//FBP2//MAPK8IP1//GNA14//CBX5//TBL1Y//KDELR1//ARF3//NDFIP2//RAB2A//XYLT2//ST8SIA2//HS3ST3B1//KIF18B//POLI//TOM1L2//TBC1D9B//TRIM44//DEPTOR//MAP1LC3A//PSMC6//ZW10//PRM1//GABPA//POU4F1//GATAD2B//ANP32E//TMEM201//AGO1//SAMD4B//GRPEL2//TXNDC8//KLHL2//FGD1//TRIM29//STARD3//CKMT2//CS//THEM5//NDUFB4//PCCB//PRODH//MAVS//NCOA4//ARPC3//SRXN1//ETF1//PDZRN3//CAMSAP2//IARS//KRT13//ARHGAP1//MTAP//PFKFB3//WWC3//PITPNM2//RANBP10//DDX55//ABCD3//LHPP//CDK2AP1//PEA15//EEF1E1//EI24//CNTNAP1//WDR3//PPM1M//ZBTB46//PPP4R2//ZNF609//SPDEF//FAM32A//NR6A1//AMFR//PROX2//HLF//MYPOP//UFM1//ANKRD49//ZC4H2//ZNF490//SNAPC1//TEAD3//VDR//ZNF24//IFRD2//ERI1//ZNF682//IFI44//PIM2//STMN2//PRDM4//MORN4//METTL21A//DNAH12//CTNNA3//MAP3K9//MYO15A//SGTB//VIPAS39//TNFAIP8L2//DPF1//SNRNP27//ESRRG//MAFG//DDA1//MORC4//ZNF672//SLC25A45//ANKRD50//ZDHHC19//SLC37A4//FA2H//TMX1//IGFBP7//ADAMTSL4//MOSPD3//RNF26//SCAMP2//LRG1//LZTR1//TPPP3//CLEC9A//VANGL1//FAM206A// |
| GO:0030424 | axon | Cellular component | 10 | 538 | 181 | 18698 | 1.92014623426236 | 0.036921041074106 | 0.511535069075114 | 1.43272606153657 | 0.0552486187845304 | MAPK8IP1//STMN2//AMFR//THY1//CNTNAP1//DNAJC5//ARPC3//CADM2//RHOA//LRP8// |
| GO:0042995 | cell projection | Cellular component | 28 | 2055 | 181 | 18698 | 1.40754661182132 | 0.0393375521786485 | 0.536364401927922 | 1.40519266801558 | 0.154696132596685 | BAIAP2//KLHL2//FGD1//DNAH12//STMN2//RHOA//NF2//CDK16//POU4F1//RIT2//VAMP2//DOC2A//ARPC3//CTNNA3//CADM2//LRP8//AMFR//FAM206A//THY1//MYO15A//MAPK8IP1//AOC3//TXNDC8//MORN4//SLC9B1//CNTNAP1//DNAJC5//C8ORF37// |
| GO:0098858 | actin-based cell projection | Cellular component | 5 | 197 | 181 | 18698 | 2.62192556861205 | 0.0429192798813063 | 0.576057209656908 | 1.36734757343019 | 0.0276243093922652 | AOC3//BAIAP2//MYO15A//NF2//MORN4// |
| GO:0005874 | microtubule | Cellular component | 8 | 408 | 181 | 18698 | 2.02556602751598 | 0.0454301169170737 | 0.60037646818102 | 1.34265614550158 | 0.0441988950276243 | KIF18B//CAMSAP2//ZW10//BAIAP2//DNAH12//TPPP3//STIM1//MAP1LC3A// |
| GO:0150034 | distal axon | Cellular component | 6 | 268 | 181 | 18698 | 2.31277315082048 | 0.0465033035872871 | 0.602999255588626 | 1.33251619380324 | 0.0331491712707182 | STMN2//AMFR//THY1//MAPK8IP1//DNAJC5//ARPC3// |
| GO:0045202 | synapse | Cellular component | 14 | 880 | 181 | 18698 | 1.64347061778001 | 0.0470550174192251 | 0.602999255588626 | 1.32739406147573 | 0.0773480662983425 | PDZRN3//BAIAP2//LRP8//CDK16//VAMP2//DNAJC5//SYT2//SLC17A5//DOC2A//RHOA//GRIA3//ZC4H2//CADM2//MAPK8IP1// |
| GO:0030658 | transport vesicle membrane | Cellular component | 5 | 203 | 181 | 18698 | 2.54443023160874 | 0.0477345161583546 | 0.602999255588626 | 1.32116747512739 | 0.0276243093922652 | HLA-DPA1//VAMP2//SYT2//SLC17A5//DOC2A// |
